# Bacterial Synthesis of Ternary CdSAg Quantum Dots through Cation Exchange: Tuning the Composition and Properties of Biological Nanoparticles for Bioimaging and Photovoltaic Applications

**DOI:** 10.3390/microorganisms8050631

**Published:** 2020-04-27

**Authors:** Nicolás Órdenes-Aenishanslins, Giovanna Anziani-Ostuni, Juan Pablo Monrás, Alejandra Tello, Denisse Bravo, Daniela Toro-Ascuy, Ricardo Soto-Rifo, Paras N. Prasad, José Manuel Pérez-Donoso

**Affiliations:** 1 BioNanotechnology and Microbiology Lab, Center for Bioinformatics and Integrative Biology (CBIB), Facultad de Ciencias de la Vida, Universidad Andrés Bello, Av. República #330, Santiago 8370186, Chile; nicolas.ordenes@gmail.com (N.Ó.-A.); giovi.anziani@gmail.com (G.A.-O.); jpmonras@gmail.com (J.P.M.); 2 Institute for Lasers, Photonics and Biophotonics, University at Buffalo, Buffalo, NY 14260, USA; 3 Laboratorio de Nanotecnología, Recursos Naturales y Sistemas Complejos, Facultad de Ciencias Naturales, Departamento de Química y Biología, Universidad de Atacama, Copayapu 485, Copiapó 1531772, Chile; atellozamorano@gmail.com; 4 Laboratorio de Microbiología Oral, Facultad de Odontología, Universidad de Chile, Sergio Livingstone Pohlhammer # 943, Santiago 8380492, Chile; denisseb@gmail.com; 5 Molecular and Cellular Virology Laboratory, Virology Program, Institute of Biomedical Sciences, Faculty of Medicine, Universidad of Chile, Independencia, Santiago 834100, Chile; daniela.toroascuy@gmail.com (D.T.-A.); rsotorifo@uchile.cl (R.S.-R.)

**Keywords:** ionic exchange, tunable nanoparticles, aqueous synthesis, nanoparticle biosynthesis

## Abstract

In this study, we introduce a biological method for the production of ternary Quantum Dots (QDs): complex nanostructures with tunable optical and structural properties that utilizes post-synthesis modifications through cation exchange. This versatile in-situ cation exchange method being reported for the first time shows great potential for extending the scope of microbial synthesis. By using this bacterial-based method, we easily synthesize and purify CdS, CdSAg, and Ag_2_S nanocrystals of a size below 15 nm and with variable morphologies that exhibit fluorescence emissions covering a broad spectral range (from 400 to 800 nm). Energy-dispersive X-ray spectroscopy (EDS) results indicate the partial replacement of Cd^2+^ by Ag^+^ when AgNO_3_ concentration is increased. This replacement produces CdSAg ternary QDs hetero-structures with high stability, fluorescence in the NIR-I (700 - 800 nm), and 36.13% quantum yield. Furthermore, this reaction can be extended for the production of soluble Ag_2_S nanoparticles (NPs) without any traces of Cd. QDs biosynthesized through this cation exchange process display very low toxicity when tested in bacterial or human cell lines. Biosynthesized ternary hetero-structures were used as red fluorescent dyes to label HeLa cells in confocal microscopy studies, which validates its use in bioimaging applications in the near infrared region. In addition, the application of biologically-produced cadmium NPs in solar cells is reported for the first time. The three biosynthesized QDs were successfully used as photosensitizers, where the CdSAg QDs show the best photovoltaic parameters. Altogether, obtained results validate the use of bacterial cells for the controlled production of nanomaterials with properties that allow their application in diverse technologies. We developed a simple biological process for obtaining tunable Quantum Dots (QDs) with different metal compositions through a cation exchange process. Nanoparticles (NPs) are produced in the extracellular space of bacterial cells exposed to cysteine and CdCl_2_ in a reaction that depends on S^2−^ generation mediated by cysteine desulfhydrase enzymes and uses cellular biomolecules to stabilize the nanoparticle. Using this extracellular approach, water-soluble fluorescent CdS, CdSAg, and Ag_2_S Quantum Dots with a tunable emission ranging from 400 to 800 nm were generated. This is the first study reporting the use of microorganisms to produce tunable ternary QDs and the first time that a cation exchange process mediated by cells is described. Obtained results validate the use of biological synthesis to produce NPs with new characteristics and opens a completely new research field related to the use of microorganisms to synthesize complex NPs that are difficult to obtain with regular chemical methods.

## 1. Introduction

Synthesis of nanoparticles using microorganisms (biosynthesis) represents a novel alternative to traditional chemical synthesis that allows economical production of biocompatible nanostructures with a tunable size and physical properties. During biosynthesis, different biomolecules interact with these in-situ produced nanoparticles (NPs) by modifying their surface and improving their stability as well as their biological function and cytotoxicity [1,2].

Even when the utilization of a variety of eukaryotic and prokaryotic organisms to generate different metal nanoparticles, like Au and Ag, was reported [3,4,5,6], little is known about the process of microbiological synthesis of semiconductor nanoparticles such as Quantum Dots (QDs). QDs are an important type of inorganic nanoparticles for numerous technological applications [7].

QDs biosynthesis is a more complex process than biological synthesis of metal nanoparticles (e.g., Cu^0^, Te^0^, Ag^0^) because the formation of nanostructures with multiple compositions requires the interaction of elements in a certain oxidation state (e.g., Cd^2+^, S^2−^, Te^2−^, Ag^+^). In general, most protocols for CdS QDs biosynthesis in bacteria involve thiols and/or metal binding cysteine-rich molecules such as phytochelatins and metallothioneins [8,9]. Cellular thiols provide a source of S for CdS and also act as stabilizing agents coating the nanoparticle [10,11]. Some sulfate reducing bacteria (such as *Desulfovibrio desulfuricans*) or enterobacteria (such as *Salmonella enterica* serovar Typhimurium) can reduce sulfate or thiosulfate to produce H_2_S [12,13]. Our laboratory has previously reported that metabolically-produced H_2_S is responsible for the formation of CdS and CdTe QDs at a low temperature [14,15]. Although *E. coli* does not have these systems, H_2_S is generated in the presence of L-cysteine as a byproduct of cysteine desulfhydrase enzymatic activity [16]. In this sense, the use of L-cysteine as a H_2_S precursor in *E. coli* cells and the use of a purified cysteine desulfhydrase enzyme for the biological synthesis of CdS nanoparticles have been recently reported [17,18].

The number of publications describing the biosynthesis of Cd-based QDs has increased in recent years. However, the application of biosynthesis processes for a precise control of QDs properties, or the use of microorganisms to produce QDs with novel compositions have been scarcely reported. In this context, the importance of phosphorylated molecules to control the characteristics of Cd-based QDs synthesized by *E. coli* has been demonstrated [19]. In addition, in recent years, the few publications that have reported the use of biosynthesis to generate QDs with improved properties described an increased biocompatibility. Nevertheless, recently, our group published the use of extremophile acidophilic microorganisms to synthesize CdS QDs with high stability at acidic conditions [20] and the use of polyextremophile halophilic bacteria to produce QDs with increased stability under osmotic stress conditions [21]. This biosynthetic process is highly versatile and has been recently used for the controlled production of CdS/CdSe Core/Shell QDs. These Cd-based nanomaterials were specifically designed for photovoltaic applications and their use as “bio-sensitizers” in QDs-sensitized solar cells was reported for the first time [22]. Based on this and the inherent advantages of using microorganisms as nanocrystal bio-factories, we believe that the biosynthesis process constitutes a unique opportunity to generate nanoparticles with improved properties, such as ternary QDs, that could contribute to the development of new technologies and applications.

Ternary Quantum Dots (QDs) such as HgCdTe, CdZnS, or CdSAg are vital for the manufacturing of new multifunctional materials and for obtaining a greater control of the optoelectronic properties of NPs [23,24,25]. The addition of metals such as Mn^2+^, Zn^2+^, and Ag^2+^, among others, is used to modify the properties of nanoparticles such as growth rate, photoluminescence, size, and crystal phase [26,27]. The variation in the composition of ternary QDs generates hetero-structures with desirable properties for biomedical applications, such as increased tissue penetration, less interference (auto-fluorescence), and reduced photochemical damage [28,29].

While methods for *de novo* synthesis of NPs have advanced considerably, post-synthesis modifications enabling the development of complex materials such as ternary QDs are scarce. Among these methods, cation exchange is a useful and versatile tool for controlling the composition and properties of synthesized NPs [30,31]. In this process, cations forming the nanocrystal (e.g., Cd^2+^ in CdS) are replaced by those in solution (e.g., Ag^+^ in AgNO_3_ solution), which allows the generation of complex hetero-structures through a delicate control of the NP composition [32]. Cation exchange is useful for the synthesis of complex nanostructures and, to date, no reports of biomimetic or biological methods to synthesize NPs involving cation exchange have been published.

In the present work, we describe a new biological synthesis method to obtain water dispersible fluorescent CdS, CdSAg, and Ag_2_S nanoparticles using *E. coli* cells. Results indicate the partial replacement of Cd^+2^ by Ag^+^ when biosynthesized CdS QDs are exposed to AgNO_3_ concentrations. This is a process that requires the presence of living cells and occurs through a cation exchange mechanism.

## 2. Materials and Methods

### 2.1. Bacterial Strains and Stock Solutions

The bacterial strain used in all experiments was *Escherichia coli* BW25113. All stock solutions were prepared in Type I Ultrapure water (Winkler, Santiago, Chile).

### 2.2. Extracellular Biosynthesis of QDs

*E. coli* was grown in an LB culture medium at 37 °C under 180 rpm agitation. After reaching an exponential phase (OD_600_ ~ 0.6), cells were washed twice with Borax-Citrate buffer 15 mM (pH 9.4) and then suspended in buffer supplemented with 1.0 mM cysteine and 100 µM CdCl_2_ (Sigma-Aldrich, St. Louis, MO, USA). Cultures were left in a 37 °C shaker. Aliquots were obtained after different time intervals, centrifuged to discard bacterial cells (15 min at 13.000 × *g*), and the fluorescence of supernatants was evaluated.

For the biosynthesis of CdSAg and Ag_2_S QDs, right after the addition of CdCl_2_ and cysteine, different concentrations of AgNO_3_ (Sigma-Aldrich, St. Louis, MO, USA) were added to the suspended cells. The AgNO_3_ concentrations used for the experiments were between 15 to 200 µM. Then, the cultures were centrifuged for 15 min at 13.000 × *g* and the NPs were recovered from the supernatant.

### 2.3. Evaluation of Cell Extracts to Produce QDs

*E. coli* was grown in LB culture medium at 37 °C with constant agitation (180 rpm). After reaching an exponential phase (OD_600_ ~ 0.6), cells were washed twice with Borax-Citrate buffer 15 mM (pH 9.4). After that, the cells were homogenized three times using glass beads and the FastPrep-24™ Classic (MP Biomedicals, LLC, Irvine, USA) for 1 min at 4 m/s. Subsequently, the samples were centrifuged for 3 min at 27,670 x g and the synthesis of nanoparticles was evaluated in the conditions described above. Protein concentration of the lysate was determined by a Quick Start™ Bradford Protein Assay (Bio-Rad Inc., Hercules, CA, USA).

### 2.4. Purification and Concentration of Extracellular Nanoparticles

Culture supernatant containing the nanoparticles was filtered through 0.22-µm filters (Jet Bio- Filtration Co., Guangzhou, China) to eliminate the cells and other particles. Purification and concentration of the filtered NPs was obtained by ultracentrifugation with 3 kDa Amicon^®^ Ultra filters (Merck Millipore, Burlington, VT, USA) according to manufacturer’s instructions.

### 2.5. Characterization of the Nanoparticles

#### 2.5.1. SEM and Elemental Analysis (EDS)

Purified CdS, CdSAg, and Ag_2_S NPs samples were coated with gold and then visualized by scanning electron microscopy at 20 kV on a Hitachi SU70SEM FESEM. The elemental analysis of the biosynthesized NPs was made on the same equipment using an Energy-dispersive X-ray Spectrometer (EDS) detector.

#### 2.5.2. Spectroscopic Properties

Absorbance and fluorescence spectra of purified nanoparticles produced by bacteria were measured by using a multiplate reader Synergy^TM^ H1 (BioTek Instrument Inc, Winooski, VT, USA). Emission spectra were obtained after excitation at 365 nm and recorded in the range of 400–800 nm.

#### 2.5.3. Quantum Yield (QY)

The following procedure of Quantum Yield (QY) determination was applied to purified CdS and CdSAg NPs solved in distilled water, and also for Nile blue in ethanol (QY = 0.27) (Sigma-Aldrich, St. Louis, MO, USA). Different samples with absorbance values between 0.01 and 0.1 A.U. under excitation at 360 nm were prepared. Fluorescence spectra were recorded for obtaining the integrated fluorescence intensity (IFI). Then, IFI was plot versus the absorbance of NPs’ solutions. Curves’ slopes (*m*) and refractive index of the solvent (*n*) (water: 1.333, and ethanol: 1.335) were used to calculate NPs QY by considering Nile blue as reference (*R*), using the following equation: *QY_NPs_ = QY_R_[m_NPs_/m_R_][n^2^_NPs_/n^2^_R_]^−1^* [19].

#### 2.5.4. X-ray Diffraction (XRD)

XRD patterns were obtained using a Bruker D8 Advance model and Lynxeye detector. Cu-Kα Radiation (30 mA, 40 KV, λ = 1.54 Å) had a scanning range of 10°−80° and step of 0.002°.

#### 2.5.5. High-Resolution Transmission Electron Microscopy (HR-TEM)

High-resolution transmission electron microscopy (HR-TEM) measurements were made using a JEOL JEM 2010, operated at 200 kV. For these studies, a drop of the dispersed sample was left to dry out on a commercial coal formvar Cu TEM grid 300 mesh copper grid hole with a size of 63 µm. HRTEM images were processed and analyzed with Digital Micrograph 3.9.0 (Gatan Inc.) and the Gimp 2.4.0 software packages. A statistical study of HRTEM images was carried out in order to quantify the particle size by measuring the diameter of 150 particles for each sample. Counts were then plotted as frequency histograms and the mean particle size was calculated. Chemical characterization of the samples was performed by Energy-dispersive X-ray spectroscopy (EDS o EDX) and ED (electron diffraction).

#### 2.5.6. Fourier Transform Infrared Spectroscopy (FT-IR)

Biosynthesized QDs were lyophilized and the obtained powder was mixed with KBr (Sigma-Aldrich, USA). Obtained KBr pellets were measured in a solid FT-IR spectrometer between 4000 and 400 cm^−1^, using a Spectrum Two FT-IR Spectrometer (PerkinElmer Inc., Waltham, MA, USA).

### 2.6. Toxicity of Biosynthesized Nanoparticles

#### 2.6.1. MTS Cell Proliferation Assay

To assess the cytotoxicity of NPs on eukaryotic cells, an MTS (3-(4,5- dimethylthiazol-2-il)-5-(3-carboxymethoxyphenyl)-2-(4-sulfophenyl)-2H-tetrazolium) (Promega Co., Madison, WI, USA) assay was performed in AGS human gastric cancer cells. A total of 20,000 cells per well were incubated for 24 h at 37 °C and 5% CO_2_ to allow adherence of cells to the plate. After 24 h, cells were treated with different concentrations and dilutions of NPs. Again, treated cells were incubated for 24 h at 37 °C and 5% CO_2_. Then, cells were washed with PBS (3 times) and treated with 100 mL of a mixture of phenazine methosulfate and MTS regarding the ratios indicated by the manufacturer (Promega Co., Madison, WI, USA). After 1 h of incubation, the absorbance at 490 nm was measured.

#### 2.6.2. Effect of Biosynthesized QDs on Bacterial Growth

The effect of biosynthesized QDs on *E. coli* growth was evaluated following the protocol previously described [33]. Briefly, *E. coli* cultures (OD_600_ ∼ 0.15) were incubated at 37 °C with agitation in the presence of QDs (100, 300, and 500 μg/mL) and turbidity was measured using a Unico 2800 UV-vis spectrophotometer.

### 2.7. Fluorescence Microscopy

HeLa cells were cultured in 12-well plates with covers glass at 100.000 cells/well and transfected with 25 μL of polyethylenimine (PEI) (PolySciences Inc., Warrington, USA) at 300 mg/L and 50 μL CdSAg at 10 mg/mL. As treatment controls, only the PEI or only the CdSAg were used. At 24 h post transfection, cells were washed twice with 1X PBS and fixed for 10 min at room temperature with 4% paraformaldehyde (Sigma-Aldrich, St. Louis, MO, USA). Then, cells were washed three times with 1X PBS. Lastly, nuclei were labeled for 1 min with a 0.3 μg/mL solution of 4′, 6-diamino-2-phenylindole (DAPI) (Life Technologies Co., Carlsbad, CA, USA) in PBS, at room temperature, washed three times with 1X PBS, washed three times with water, and mounted with 1,4-diazabicyclo [2.2.2] octane (DABCO) (Merck Millipore, Burlington, VT, USA). Images were obtained with a TCS SP8 Confocal Microscope (Leica Microsystems, Wetzlar, Germany) and obtained images were processed using FIJI/ImageJ (NIH). Excitation was 555 nm and depletion was 660 nm.

### 2.8. Fabrication and Characterization of Quantum Dot Sensitized Solar Cells (QDSSC)

QDSSCs were produced following the protocol previously described by our group [34] with some modifications. To fabricate the electrodes, 20 × 20 × 2 mm in size fluorine-doped tin oxide coated glass (FTO glass) TEC15 with a surface resistivity of 13 [Ω/sq] and 85% transmittance was used (Sigma-Aldrich, St. Louis, MO, USA). Conductive glasses were cleaned by successive sonication in absolute ethanol and deionized water for approximately 10 min to remove organic contaminants. The anode was prepared using a suspension of titanium (IV) oxide nanoparticles (TiO_2_ Nano powder ~21 nm particle size and anatase crystal structure, Sigma-Aldrich USA) that was deposited on the glass by spin-coating at 2000 rpm for 10 s. TiO_2_ film underwent a sintering process at 465 °C for 20 min and sensitization was performed by direct adsorption. The electrode active area (1 cm^2^) was treated once with 50 mg/mL NP solution and incubated in darkness. The cathode or counter electrode was prepared as previously described [34]. Then, the photoanode and the counter electrode were assembled by leaving a 127-μm space between them. Before sealing the cell, 7 μL of electrolyte sulfide/polysulfide (S^2−^/S*_n_*^2−^) solution was added (Na_2_S 1.0 M, S 0.1 M, NaOH 0.1 M, from Sigma-Aldrich, USA, in ultrapure water from Winkler Ltd.a., Chile). Characterization of solar cells was performed in triplicate for each NP using a solar simulator (A1 Solar LightLine, Sciencetech Inc., ON, Canada) and a current-voltage measurement system (IV Tester- 20W, Sciencetech Inc., ON, Canada). Measurements were performed under constant conditions of temperature and irradiance at a one sun intensity as the light source (~100 mW·cm^−2^ and AM1.5).

## 3. Results

### 3.1. Biosynthesis of CdS QDs in E. coli

**Figure 1:**
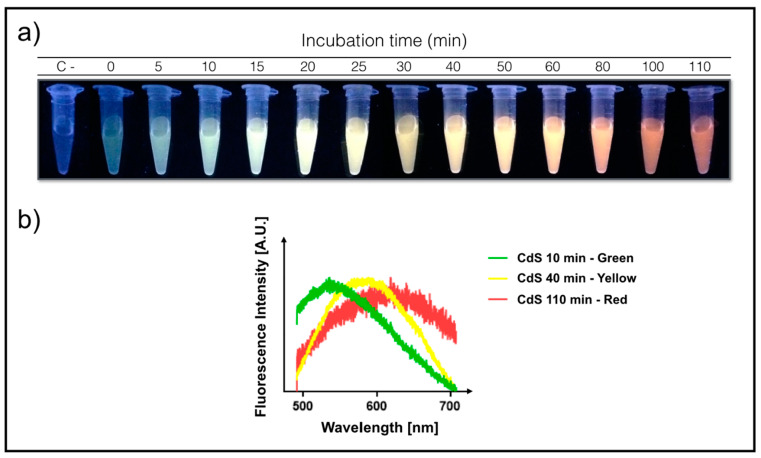
(**a**) Kinetics of extracellular biosynthesis of CdS Quantum Dots (QDs). Ultra Violet-exposed supernatants of bacterial cultures at different incubation times with cadmium and cysteine (λ_exc_ = 365 nm). (**b**) Fluorescence emission spectra of the three most representative fractions obtained during QDs biosynthesis (λ_exc_ = 405 nm).

An intrinsic property of QDs is the fluorescence emission when excited with UV light. Based on this, bacterial synthesis of QDs can be determined by detecting fluorescence emission in cell pellets and supernatants [11,14]. *E. coli* cells at an exponential growth phase were exposed to Cd^+2^ and cysteine, and the time-dependent shift in NPs growth was determined by measuring QDs-associated bacterial fluorescence in cell cultures exposed to UV light. Furthermore, different fluorescence emission colors were observed in culture supernatants (extracellularly) at different incubation times, which changed from green to yellow and then to red (Figure 1a). CdS nanoparticles obtained by extracellular biosynthesis also display different emission spectra depending on reaction time with emission profiles ranging from 500 to 700 nm (Figure 1b). This is a characteristic behavior of QDs associated with the increase of NPs’ size [14,19].

### 3.2. Biosynthesis of Ternary QDs in E. coli

Some chemical methods to synthesize CdS QDs involve the addition of metals as dopants to modify the properties of NPs and generate complex nanostructures [26,27,35]. To evaluate the effect of Ag^2+^ on CdS nanoparticles, we used AgNO_3_ as a silver source since there is evidence for this metal to help in cation exchange interactions and because CdS/Ag structures display unique optical properties [32,36,37].

**Figure 2:**
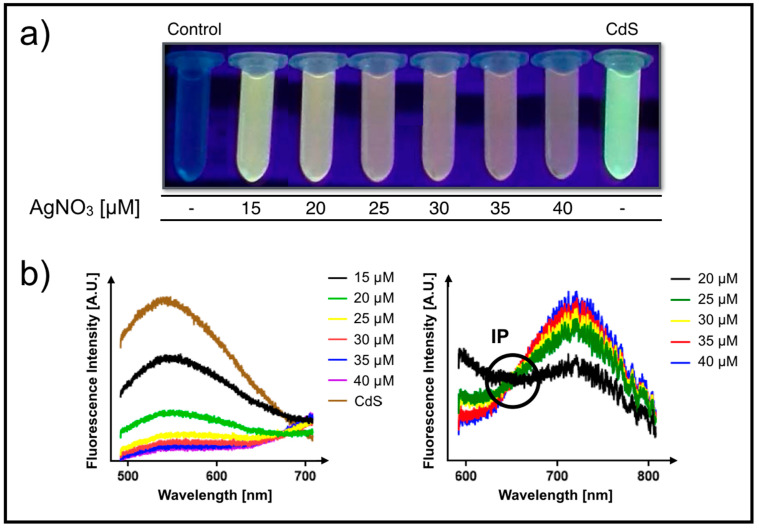
Biosynthesis of CdS Quantum Dots (QDs) in the presence of different AgNO_3_ concentrations (15 to 40 µM). Negative control condition contains CdCl_2_ (100 µM) in the absence of cysteine (no fluorescence). The positive control (CdS) was conducted without the addition of AgNO_3_. (**a**) Biosynthesis solutions obtained in the presence of different AgNO_3_ concentrations exposed and not exposed to UV. Concentrations of reagents used in each reaction are indicated. (**b**,**c**) Fluorescence spectra of samples containing AgNO_3_ at different concentrations. The isosbestic point (IP) is also shown.

When increasing Ag^+^ concentrations in the extracellular biosynthesis reaction (AgNO_3_ 15 to 40 µM), the spectroscopic properties of CdS-QDs present in bacterial supernatants changed (Figure 2a). Silver-treated solutions (none-exposed to UV light) display significant changes in color, changing from uncolored to a red color after exposure to AgNO_3_ 40 μM, or to a dark gray when AgNO_3_ 200 μM was used in the reaction (Figure 2b). The emission of the nanoparticles produced in the presence of Ag^+^ is red shifted with emissions between 650 and 800 nm as compared to CdS ranging from 500 to 700 nm (Figure 2c). This shift in the fluorescence spectra forms a static point that does not change in all concentrations of AgNO_3_ tested, called an isosbestic point (IP). The existence of an IP indicates that the reaction involves the conversion of one species into a unique other species, or that an equilibrium between only two species is reached. It certainly shows that the stoichiometry of the reaction remains unchanged during the chemical reaction and that no secondary reactions occur during the considered time range.

### 3.3. Characterization of Biosynthesized Ternary QDs

**Figure 3:**
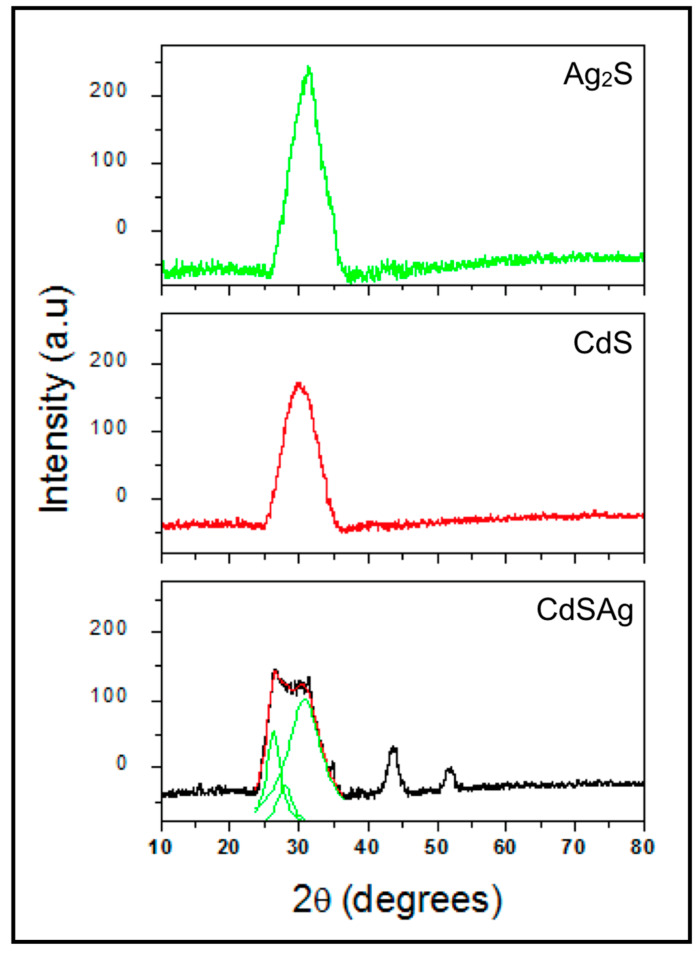
X-ray diffractograms of nanoparticles biosynthesized by *E. coli*, for Ag_2_S, CdS and CdSAg purified nanoparticles.

NPs biosynthesized under three selected conditions (0, 40, and 200 μM AgNO_3_) were purified and characterized. As the first characterization, XRD analyses were performed for the three purified QDs. As shown in Figure 3, wide diffraction peaks were observed in all samples. According to the Scherrer equation and assuming homogeneous tension, these peaks are consistent with the characteristic pattern of nanoparticles or small crystallites (Figure 3) [38]. In the case of CdS-NPs, a single diffraction plane at (200), corresponding to the Hawleyite mineral phase as derived from the CPDS pattern, was observed (Figure 3, CdS) [39]. NPs produced in the presence of 200 μM AgNO_3_ (Figure 3, Ag_2_S) shows a single diffraction peak in the crystallographic plane (111), corresponding to the CPDS pattern of the Argentite cubic phase, Ag_2_S [40]. These results indicate that CdS and Ag_2_S NPs have a preferential orientation for crystalline growth. When 40 μM AgNO_3_ was incorporated in the biosynthesis solution, more than one diffraction peak was observed, which suggests the formation of a ternary structure (Figure 3, CdSAg). Since increased noise from the diffraction peak between 20 and 35° indicate more than one signal or fusion of multiple diffraction peaks, deconvolution, and Lorentzian curves adjustment were performed. From these adjustments, it is assumed that the diffraction peak for the first Lorentzian correspond to a crystalline plane (111), while the second one corresponds to a CdS crystalline plane (200), which is congruent with the Hawleyite phase of the CPDS pattern. For the third Lorentzian, the diffraction peak adjustment for the crystalline plane (111) of Ag_2_S is congruent with the Argentite phase CPDS pattern. Additionally, diffraction peaks at (220) and (113) were observed, which correspond to the CdS Hawleyite phase CPDS pattern (Figure 3, CdS).

**Figure 4:**
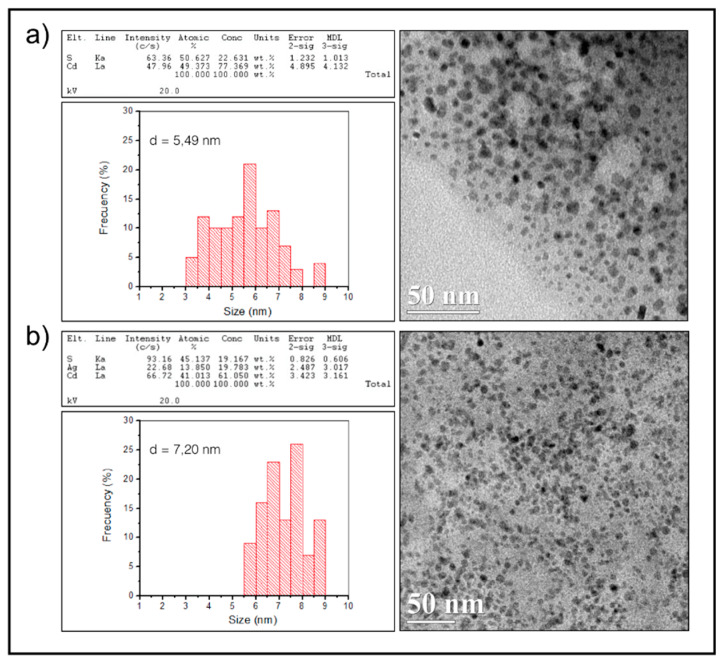
EDS, HR-TEM, and frequency histograms of biosynthesized CdS (**a**) and CdSAg (**b**) nanoparticles.

Biosynthesized NPs were then analyzed by High-Resolution Transmission Electron Microscopy (HR-TEM). As shown in Figure 4, regular polyhedral nanometric structures were observed for CdS and CdSAg NPs (Figure 4a,b). The NPs’ average diameter was determined from frequency histograms, corresponding to 5.49 nm for CdS NPs and 7.20 nm for CdSAg NPs (Figure 4a,b).

The energy-dispersive X-ray spectroscopy (EDS) analysis for the CdS NPs indicated that Cd and S elements are present with an atomic proportion of 50% and 49%, respectively (Figure 4a). Additionally, the EDS analysis of the ternary NPs produced in the presence of 40 μM AgNO_3_ indicated the presence of Cd, S, and Ag elements with an atomic proportion of 41.013%, 45.137%, and 13.850%, respectively (Figure 4b).

Quantum yield (QY) values of fluorescent nanoparticles (0 and 40 μM AgNO_3_) were determined. The obtained results indicated QYs of 20.3% for CdS and 36.13% for CdSAg QDs. Regarding ternary QDs biosynthesis, this is the maximum quantum yield reported for biologically produced nanostructures, which is a condition that reinforces the importance and novelty of this cell-based synthesis method, particularly considering their potential applications.

Most biologically produced nanomaterials display an external layer constituted by a variety of biological molecules that could affect their properties. To evaluate if the different Ag concentrations used for the biosynthesis of ternary QDs affects the organic components present in biosynthesized NPs, an FT-IR analysis was performed. Identical FT-IR spectra were determined for CdS, CdSAg, and Ag_2_S nanoparticles (Figure S1). In all of them, the presence of signatures from the O-H stretch (3000–3700 cm^−1^), aromatic C=C (1700 cm^−1^), C=O (1500–1650 cm^−1^), N-H bend (1250–1450 cm^−1^), and aliphatic amines (950–1100 cm^−1^) molecules were determined (Figure S1). All these signals can be attributed to the presence of biomolecules produced by *E. coli* and bound to the nanoparticles (i.e., proteins or peptides, coenzymes). These biomolecules can provide support for the nucleation of the nanoparticles, and/or be involved in the biosynthesis process acting as stabilizing and capping agents [11,41,42].

Since one of the major problems that affect QDs’ applications is their poor biocompatibility, we decided to evaluate the toxicity of NPs biosynthesized by our method. No effect of biosynthesized QDs on the growth of *Escherichia coli* was determined in cultures exposed to 100-500 μg/mL NPs concentrations (Figure S2). Despite chemical differences in the nanocrystal core, no significant differences on bacterial toxicity were observed between CdS, CdSAg, and Ag_2_S NPs (Figure S2). This result is most likely a consequence of the similar capping composition present in the different NPs. In terms of eukaryotic cells, the biocompatibility of CdSAg was tested in human gastric cells (AGS). No toxicity was observed up to 50 μg/mL of NPs (Figure S3). However, a significant effect on viability was observed when AGS cells were exposed to 100 μg/mL of CdSAg NPs.

**Figure 5:**
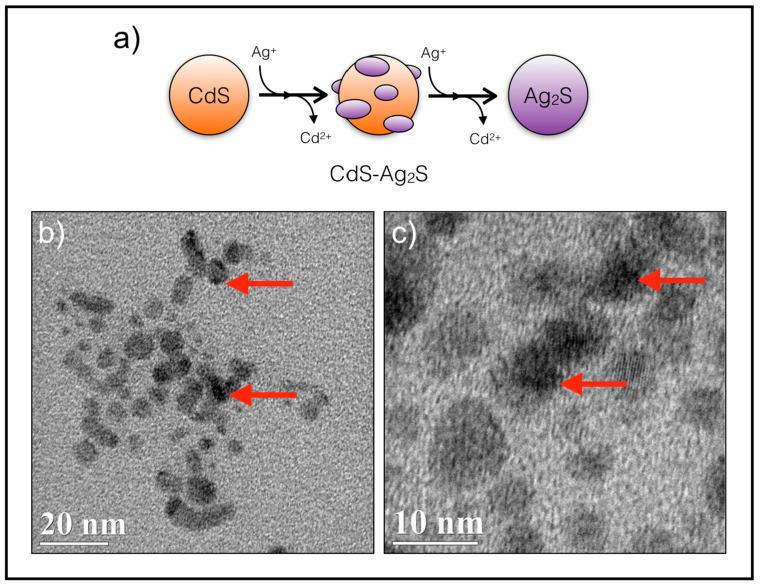
HR-TEM analysis of CdSAg biosynthesized nanoparticles. (**a**) Scheme of the formation of binary (CdS, Ag_2_S) and ternary (CdSAg) quantum dots by cation exchange. (**b**) and (**c**) Purified CdSAg NPs. Arrows indicate electrodense zones present in the ternary nanostructure.

The unique conditions and characteristics of the biosynthetic process, and the properties determined in the as-produced nanomaterials, suggest that the formation of CdSAg hetero-structures is a consequence of a cation exchange reaction between Cd^2+^ and Ag^+^ in the CdS ionic crystal [25,32]. In Figure 5b,c, HR-TEM image magnifications show the presence of electron dense regions in CdSAg NPs. If a cation exchange reaction occurs, an increase in the amount of Ag^+^ would tend to exchange all the Cd^2+^ from the NPs, eventually forming Ag_2_S. As presented in the scheme in Figure 5a, biological nanoparticles are obtained in three consecutive steps. First, nanometer sized CdS crystals are formed. These NPs present high fluorescence emission in the visible range. Then, as Ag^+^ ions are added to the reaction, an intermediary structure between CdS and Ag_2_S is produced. Lastly, the addition of a higher AgNO_3_ concentration pushes the reaction toward the formation of Ag_2_S NPs.

### 3.4. Ternary QDs Biosynthesis Requires Live E. coli Cells to Occur

**Figure 6:**
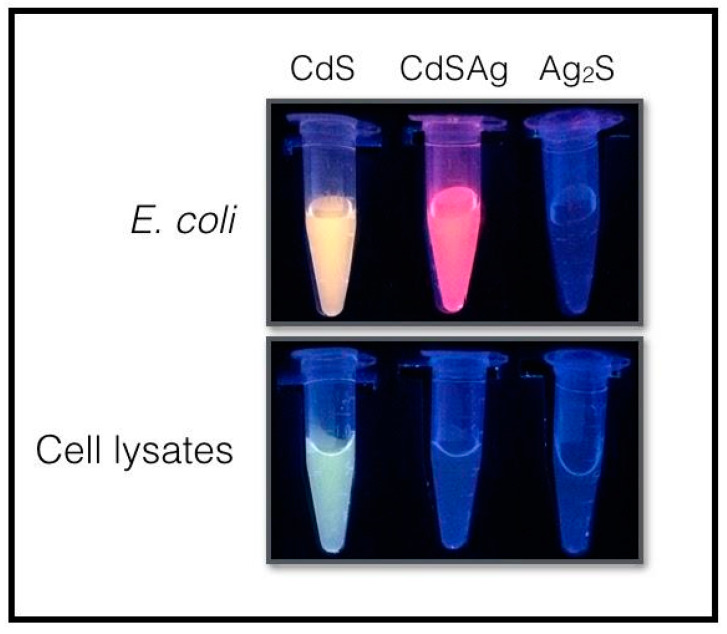
The synthesis of ternary cadmium/silver sulfide Quantum Dots requires living *E. coli* cells to occur. QDs synthesis reactions were performed in the presence of *E. coli* cells or cell lysates using the described CdS biosynthesis conditions (Cd^2+^ and cysteine). AgNO_3_ 40 or 200 μM was used to produce CdSAg or Ag_2_S NPs, respectively. Fluorescence was evaluated by exposing the tubes to ultraviolet (UV) light (λ_exc_ = 365 nm).

One of the most interesting aspects of this synthesis process is that the formation of crystalline heterostructures is conditioned to the presence of living *E. coli* cells (no fluorescence was observed in the presence of dead cells, not shown). To test if the cation exchange reaction can occur in the presence of cellular extracts, *E. coli* cell lysates were exposed to biosynthesis conditions (Cd^2+^ and cysteine). As shown in Figure 6, fluorescence was observed when both cell lysates or living cells were exposed to biosynthesis conditions, indicating that both conditions produce QDs and render effective CdS nanocrystal seeds. As expected, when AgNO_3_ (40 μM) was added to the solution containing CdS QDs in the presence of bacterial cells, a red shift in fluorescence associated with ternary QDs production was observed. When 200 μM of AgNO_3_ was added, no fluorescence was determined (as expected for Ag_2_S NPs that are not fluorescent when exposed to UV light). On the other hand, when AgNO_3_ was incorporated to CdS QDs in the presence of cell lysates, no fluorescence was observed (Figure 6). This result indicates that living cells are required for the cation exchange process to occur in a reaction that likely involves active metabolic processes, a constant production of sulfide, and/or the generation or presence in its active form of different biomolecules (reduced thiols or proteins) among others.

### 3.5. Use of Ternary NPs in Bioimaging Applications

**Figure 7:**
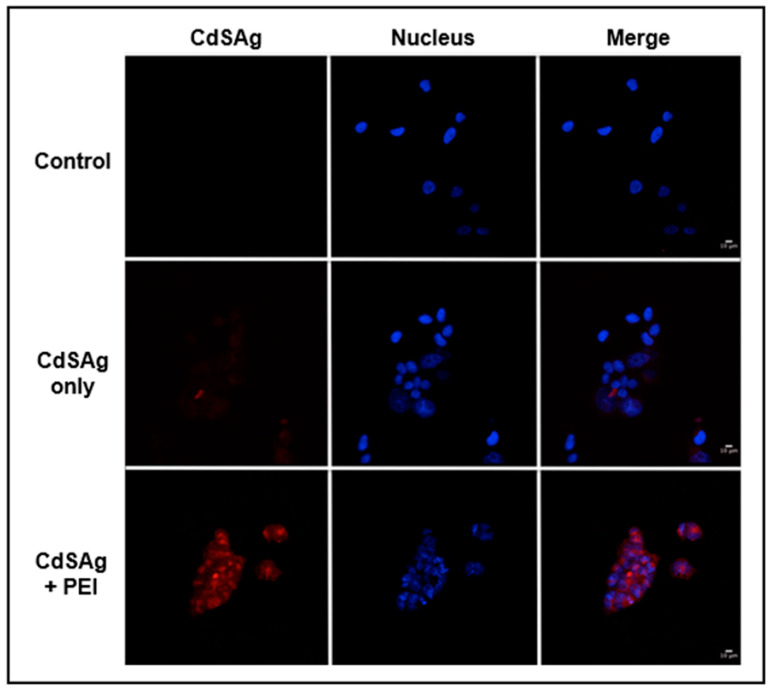
Use of biosynthesized CdSAg Quantum dots for labeling HeLa cells. HeLa cells were transfected with PEI and CdSAg QDs. Controls cells were treated only with PEI or CdSAg. Nuclei were labeled with DAPI. Images were obtained with a TCS SP8 Confocal Microscope and images were processed using FIJI/ImageJ (NIH).

As described above, ternary NPs produced by our biological cation exchange method display high biocompatibility and excellent spectroscopic properties such as high Quantum Yield and near-IR emission. These characteristics are highly desired when using fluorescent molecules to visualize biological samples (bioimaging applications). Ternary QDs were used to label HeLa cells that were co-labeled with DAPI (Figure 7). Based on previous results using biological and biomimetic Cd-QDs for labeling cells, the use of polyethylenimine (PEI) as a transfection agent is strongly suggested [43]. Additionally, in the absence of PEI, a small amount of red fluorescent cells was observed. When the transfection agent was incorporated, a high level of red labeling occurs all over the cytoplasm of HeLa cells, which validates the use of these biological QDs in bioimaging applications.

### 3.6. Use of Biosynthesized NPs as Sensitizers in Quantum Dot Sensitized Solar Cells (QDSSCs)

Based on the optoelectronic properties and QY values of the biosynthesized nanoparticles, we decided to evaluate the function of biologically synthesized Cd-based QDs in Quantum Dot Sensitized Solar Cells (QDSSCs). In this solar cell, a film of TiO_2_ nanoparticles deposited on a conductive glass is sensitized with QDs forming the photoanode, while the cathode or counter electrode is platinum on glass. Obtained photovoltaic parameters of solar cells sensitized with each of the QDs biosynthesized through the cation exchange process are summarized in Table 1 (IV curves are shown in Supplementary Figure S4).

The solar cells sensitized with ternary QDs showed the highest values compared to those cells sensitized with CdS or Ag_2_S. The biosynthesized hetero-structure generated a V_OC_ of 279 mV, I_SC_ of 0.169 mA cm^−2^, and efficiency of 0.0222%. As expected, the efficiency of the solar cell produced with ternary QDs was four times higher than the solar cell sensitized with Ag_2_S NPs and eight times higher than the one sensitized with CdS QDs.

Obtained photovoltaic parameters validate the use of bacterially-produced QDs in QDSSCs and represent an interesting projection of the present work.

## 4. Discussion

In recent decades, the production of nanomaterials has been a topic of increasing interest because of their multiple technological applications. As a consequence, legal regulations for the production, application, and safety of nanomaterials have been continuously evolving and the industrial interest has oriented to the use of eco-friendly and sustainable production methods. In this context, the use of biological methods to synthesize nanomaterials has become an interesting alternative.

In general, synthesis of nanostructures using microorganisms as reaction mediators is an eco-friendly alternative to classical chemical methods. Faster synthesis times, lower production costs, and the possibility to include new metals that allow post-synthesis modifications are some benefits of choosing this method [1,2]. However, several improvements in terms of reproducibility, versatility, and scalability of biosynthesis procedures are still required. To date, the potential of biological methods for controlling the properties and stability of NPs has been scarcely studied. Recent studies have reported the use of different microorganisms as bio-factories to produce nanoparticles with novel properties [42,44]. Examples of this are extremophile acidophilic bacterium that produces QDs resistant to acidic conditions, salt stable QDs produced by halophilic microorganisms, low temperature biosynthesis using psychrotolerant Antarctic bacteria, the biosynthesis of QDs with increased biocompatibility by yeasts, and the use of bacteria for the production of core/shell QDs for photovoltaic applications [15,20,21,22,44]. In this context, the biosynthesis of complex nanomaterials, such as binary and ternary quantum dots, or the biological production of NPs with novel and controllable properties is a scarcely explored challenge.

This work is the first report of a biological method for obtaining extracellular ternary quantum dots using bacterial cells. The described methodology produces crystalline nanostructures with tunable optical properties and offers the option of post-synthesis modifications through a cation exchange process. The extracellular QDs produced by *E. coli* cells remain in the supernatant fraction and their emission is tunable in the visible and near IR range. The biosynthesis method involves the addition of cysteine into a bacterial culture, which promotes the generation of H_2_S and the extracellular production of metal-sulfur nanoparticles [14,15,17]. The process takes advantage of bacterial enzymatic activities that release great amounts of H_2_S in the presence of cysteine. This sulfur volatile compound diffuses through the bacterial membrane and reacts with intracellular cadmium as well as with metal ions outside the cell to form CdS QDs in the extracellular domain after 30 min [15,17]. The Borax-Citrate buffer used in this method has been previously reported for the biosynthesis of CdS nanoparticles [22]. This reaction media allows the deprotonation of the H_2_S produced by the bacteria, which allows the interaction of S^2−^ with Cd^2+^ to produce the crystal [22].

In terms of the biological process involved in ternary QDs biosynthesis, we have shown that *E. coli* is able to synthesize CdS, CdSAg, and Ag_2_S NPs extracellularly when treated with CdCl_2_ and different concentrations of AgNO_3_. *E. coli* cells exposed to biosynthesis conditions metabolically produce S^2−^ and all the substrates required for nanocrystals biosynthesis including stabilizing agents such as cellular thiols, proteins, or peptides [45]. Even though CdS biosynthesis is observed with cell lysates, it is interesting that the presence of living cells is mandatory for the generation of CdSAg and Ag_2_S NPs through cation exchange reactions (Figure 6). The interaction of bacterial biomolecules is most likely helping in the stabilization of the nanoparticles outside the cell.

As mentioned above, cation exchange reactions are one of the technologies used to generate complex nanostructures such as ternary and quaternary QDs [23,30,46,47]. The method in this case allows us to biosynthesize different NPs in three subsequent steps of the same reaction, which all have different properties. The optimization of the AgNO_3_ concentration added to the reaction solution was essential in the development of this protocol. By increasing the amount of Ag^2+^ ions, we assured that CdS NPs exchange all Cd^2+^ ions by obtaining Cd-free Ag_2_S NPs. Based on this, the presence of a ternary structure was only achieved when the concentration of Ag^2+^ ions was limited by decreasing the AgNO_3_ concentration added to the reaction.

In recent years, Ag_2_S NPs have attracted considerable attention because they are used in the synthesis of other NPs such as Ag_2_S-CdS-ZnS and AgInS-Ag_2_S [48], which is due to their high biocompatibility and photoluminescent properties that makes them useful for bioimaging in deep tissues. These nanoparticles are fluorescent in the near-infrared and their emission can be tuned, depending on the nanoparticle structure and size [48]. Several protocols for the biosynthesis of silver sulfide NPs has been described with most of them involving a different process than the one described in this case since they require the incorporation of a chemical sulfur source (such as Na_2_S or Na_2_S_2_O_3_) or the sulfidation of Agº NPs. Silver sulfide cellular production has been described in bacteria (*Pseudomonas stutzeri* and *Shewanella oneidensis*) and also in HepG2 mammalian cancer cells [49,50], but no reports of cation exchange protocols for obtaining these NPs has been ever published.

Regarding the biological process involved in ternary QDs biosynthesis, we have shown that *E. coli* is able to synthesize CdS, CdSAg, and Ag_2_S NPs extracellularly when treated with different concentrations of CdCl_2_ and AgNO_3_. The presence of cysteine allows *E. coli* cells to metabolically produce S^2−^ and all the substrates required for nanocrystal biosynthesis including stabilizing agents such as cellular thiols, proteins, or peptides [45]. It is known that the use of cysteine on cell extracts or solutions stimulates the enzymatic production of sulfide that can interact with Cd^2+^ by generating fluorescent CdS NPs [18]. Even though CdS biosynthesis is observed with cell lysates, it is interesting that the presence of living cells is mandatory for the generation of NPs with a tunable size and physical properties through cation exchange reactions. In addition, the interaction of released biomolecules could help stabilize the nanoparticles outside the cell.

These “still unknown” biomolecules can control growth, polydispersity, and optoelectronic properties, among other parameters. In addition, biomolecules composing the QDs could help to decrease their toxicity in cell lines and bacteria, which has been described before [51]. In this context, no effect on bacterial growth was observed with the three NPs even at high concentrations (500 μg/mL) (Figure S2). This result is somehow surprising considering that QDs are composed by toxic elements like Cd and Ag, and also a significant effect on bacterial growth curves has been previously reported in *E. coli* cultures exposed to 500 μg/mL Cd-QD synthesized by chemical methods [51]. In human cells, we observed that CdSAg QDs does not affect viability at 50 μg/mL but generate a significant cell death at 100 μg/mL. In a previous report, working with a different kind of Cd-based QDs produced chemically, a 10% to 20% cell death after exposure to 50 or 100 μg/mL CdTe QDs was observed [43]. Toxicity could be associated with intracellular release of Cd^+2^ and/or Ag^+^, or the production of reactive oxygen species, which has been reported with other QDs [33]. However, it is important to mention that, currently, there are no studies reporting the toxicity of CdSAg QDs.

Ternary CdSAg QDs described in this case represent an intermediate structure between the seeds of CdS and the formation of Ag_2_S. CdSAg QDs biosynthesized by this method present higher quantum yield (QY) than CdS QDs, with 36.13% and 20.3%, respectively. In general, chemically synthesized nanostructures with QY values between 5%–25% for binary QDs and up to 80% for quaternary structures have been reported [45,47]. This is the highest QY value reported for biosynthesized nanomaterials, which is a property that favors the potential applications of these nanoparticles, particularly considering that biologically-obtained CdSAg hetero-structures have not been described to date.

The novel properties of biosynthesized ternary QDs such as near IR emission and high QY makes them excellent candidates for bioimaging and photovoltaic applications. Based on this, we tested CdSAg QDs as fluorescent labels on eukaryotic cells. As shown in Figure 7, HeLa cells are able to incorporate the ternary QDs when incubated in the presence of the transfecting agent PEI. The incorporation into the cells is not associated with changes in the cell morphology and the fluorescence is stable, which confirms the potential of ternary QDs as intracellular markers.

In addition, solar cells were assembled using each biosynthesized NP as sensitizer and photovoltaic parameters were determined. As expected, ternary QDs sensitized solar cells showed higher photovoltaic parameters than those obtained with binary structured NPs (Table 1). When chemically produced Cd-QDs were used as sensitizers in our solar cell system, similar photovoltaic parameters than those observed with biosynthesized CdSAg QDs were determined (V_OC_ 302 v/s 279 mV, I_SC_ 0.146 v/s 0.169 mA cm^−2^, and efficiencies of 0.0204% v/s 0.0222%, respectively). Lower values were obtained with biosynthesized CdS and Ag_2_S with 10 times the decrease in efficiency. To the best of our knowledge, there are two previous works in which the use of biologically produced QDs were tested as sensitizers in solar cells [22,52]. PbS NPs produced by an engineered strain of *Stenotrophomonas maltophilia* with a similar cysteine-mediated process than the one described in this study were used as sensitizers in QDSSCs. Authors determined some photovoltaic parameters of solar cells obtaining Voc values (0.43 V) that are consistent with those reported for solar cells sensitized with chemically-produced Pb-QDs [52]. In addition, we recently reported the biosynthesis of CdS/CdSe Core/Shell and their application in QDSSCs. Reported photovoltaic parameters are consistent with those presented in this scenario in terms of Voc and efficiencies. Interestingly, biosynthesized ternary CdSAg QDs display the same efficiency than Core/Shell QDs produced by *E. coli* (0.022%) [22].

The use of Ag NPs produced by plant extracts to improve the efficiency of solar cells has been recently published [53]. In particular, Ag NPs have been incorporated to CdS-based QDSSCs to improve their efficiency [54] by obtaining similar photovoltaic parameters to those reported in this case with efficiencies of 0.057% and 0.15% for CdS and CdSAg QDs, respectively.

The advantage of using CdSAg hetero-structures as sensitizers on QDSSCs was recently reported [55]. Solar cells based on chemically synthesized CdSAg presented higher photovoltaic parameters than those constructed using TIO_2_, CdS, or Ag_2_S [55]. This result is in agreement with the photovoltaic parameters determined in cells constructed with the NPs produced by our biological method. In addition, biologically produced QDs display similar V_O_c values than those reported with chemically-synthesized QDs (2.3-2.7 mV) [55]. The better performance of these kind of heterostructures have been associated with its capacity to decrease/suppress the electron-hole pair recombination, the utilization of a wider section of the solar spectrum (increasing the photon absorption), and a better energy level alignment [55,56].

The application of QDs in photovoltaic technologies has been hindered by the costs and the environmental impact of synthesis methods. Considering the simplicity of our green method and the possibility to control the properties of the nanomaterials produced, the presented results confirm the potential of using cells as bio-factories to produce nanomaterials required by the industry.

Our results will strongly contribute to the generation of new protocols to biosynthesize cadmium semiconductor nanoparticles using microorganisms as well as for the biosynthesis of more complex QDs such as binary (e.g., CdTe, CdSe, and Ag_2_S), ternary (e.g., CdSAg, CdHgTe), and doped nanoparticles (e.g., Mn-doped CdS). Future direction of this work is to test the ability of different nanoparticle seeds to produce complex nano-heterostructures of elements like Cu, Se, Pb, Te, and In, as they can be used for new applications and technologies including optical communication systems (as IR and UV-VIS light emitting materials), energy conversion (solar cells), or bioimaging (as fluorescent labels).

## Figures and Tables

**Table 1:** Photovoltaic parameters of quantum dot sensitized solar cells constructed with biological Quantum Dots (QDs).

Photoanode Structure (Sensitizer)	Short Circuit Current I_sc_ [mA/cm^2^]	Open Circuit Voltage V_oc_ [mV]	Fill Factor FF [%]	Efficiency Ƞ [%]
CdS	0.0238 ± 0.00459	155	65.5	0.00271
CdSAg	0.1690 ± 0.02940	279	47.2	0.0222
Ag_2_S	0.0448 ± 0.00187	209	59.1	0.00547

Characterization of solar cells was performed in triplicate for each nanoparticle. Average values ± SE are shown.

## Supplementary Materials

The following are available online at https://www.mdpi.com/2076-2607/8/5/631/s1. **Supplementary Figure S1.** FTIR spectra of biosynthesized nanoparticles: (a) CdS, (b) CdSAg, and (c) Ag_2_S. **Supplementary Figure S2.** Effect of different concentrations of biosynthesized QDs on *E. coli* growth curves. **Supplementary Figure S3.** Effect of different concentrations of CdSAg QDs on the viability of AGS45 cells (MTS assay). **Supplementary Figure S4.** Current- voltage curves for solar cells constructed with biosynthesized CdS, CdSAg, and Ag_2_S NPs as a photosensitizer.
