# Supplementary figures and images for: Bacterial Synthesis of Ternary CdSAg Quantum Dots through Cation Exchange: Tuning the Composition and Properties of Biological Nanoparticles for Bioimaging and Photovoltaic Applications
Source: Microorganisms. 2020 Apr 27;8(5):631. doi: 10.3390/microorganisms8050631 (PMC7284518; doi:10.3390/microorganisms8050631)

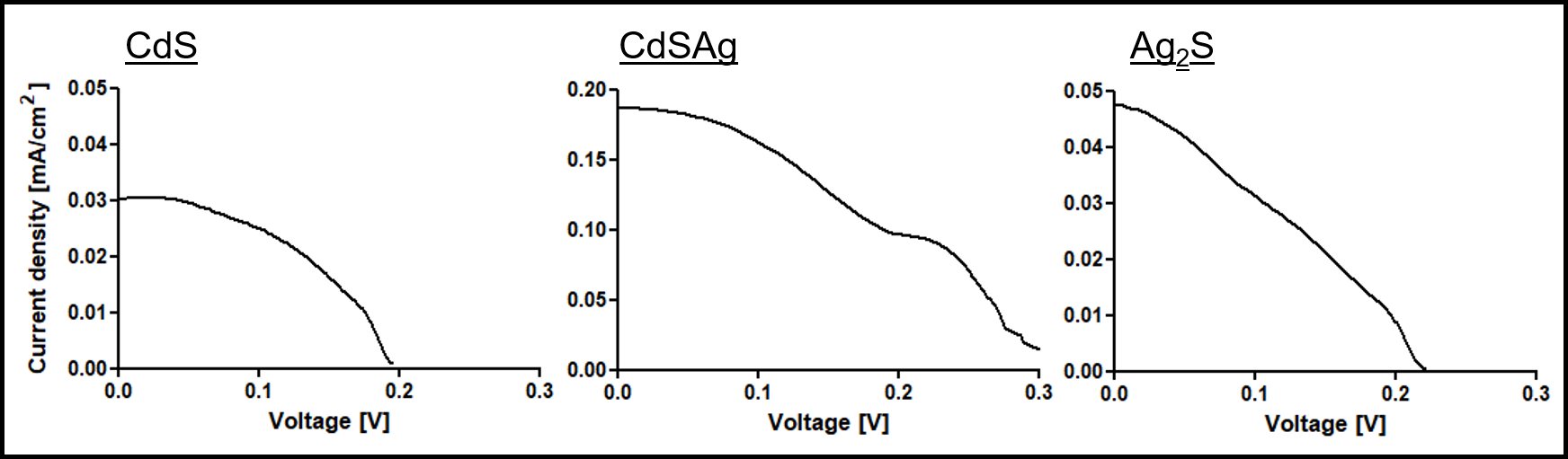

Supplement: Supplementary file 1 [file microorganisms-08-00631-s001.zip › Figura sup 4 .tif]

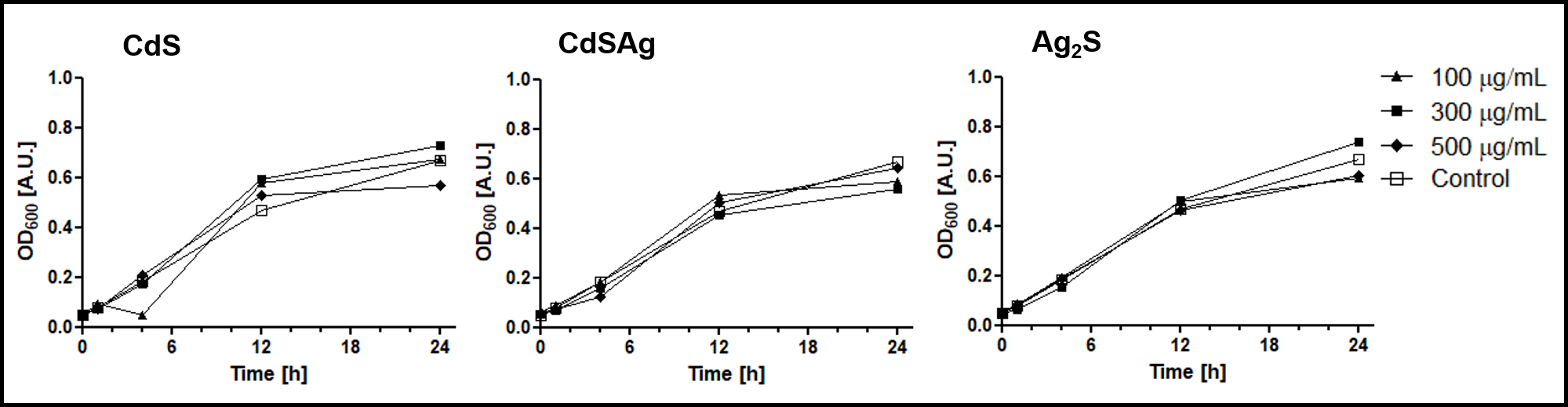

Supplement: Supplementary file 1 [file microorganisms-08-00631-s001.zip › Figura sup 2 .tif]

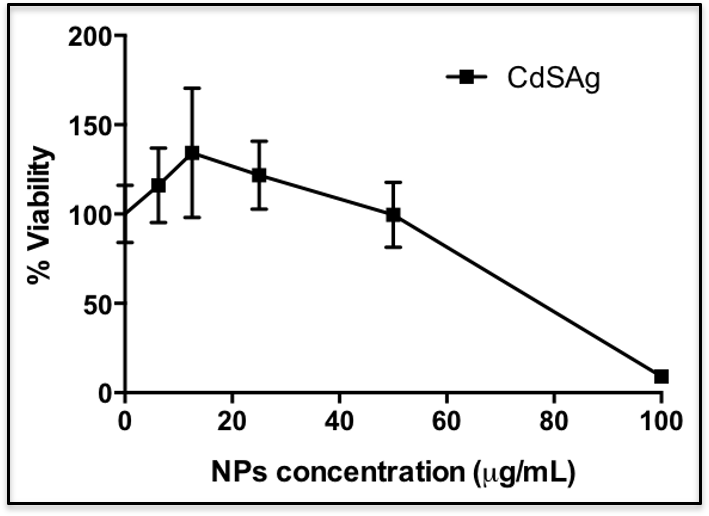

Supplement: Supplementary file 1 [file microorganisms-08-00631-s001.zip › Figura sup 3.tif]

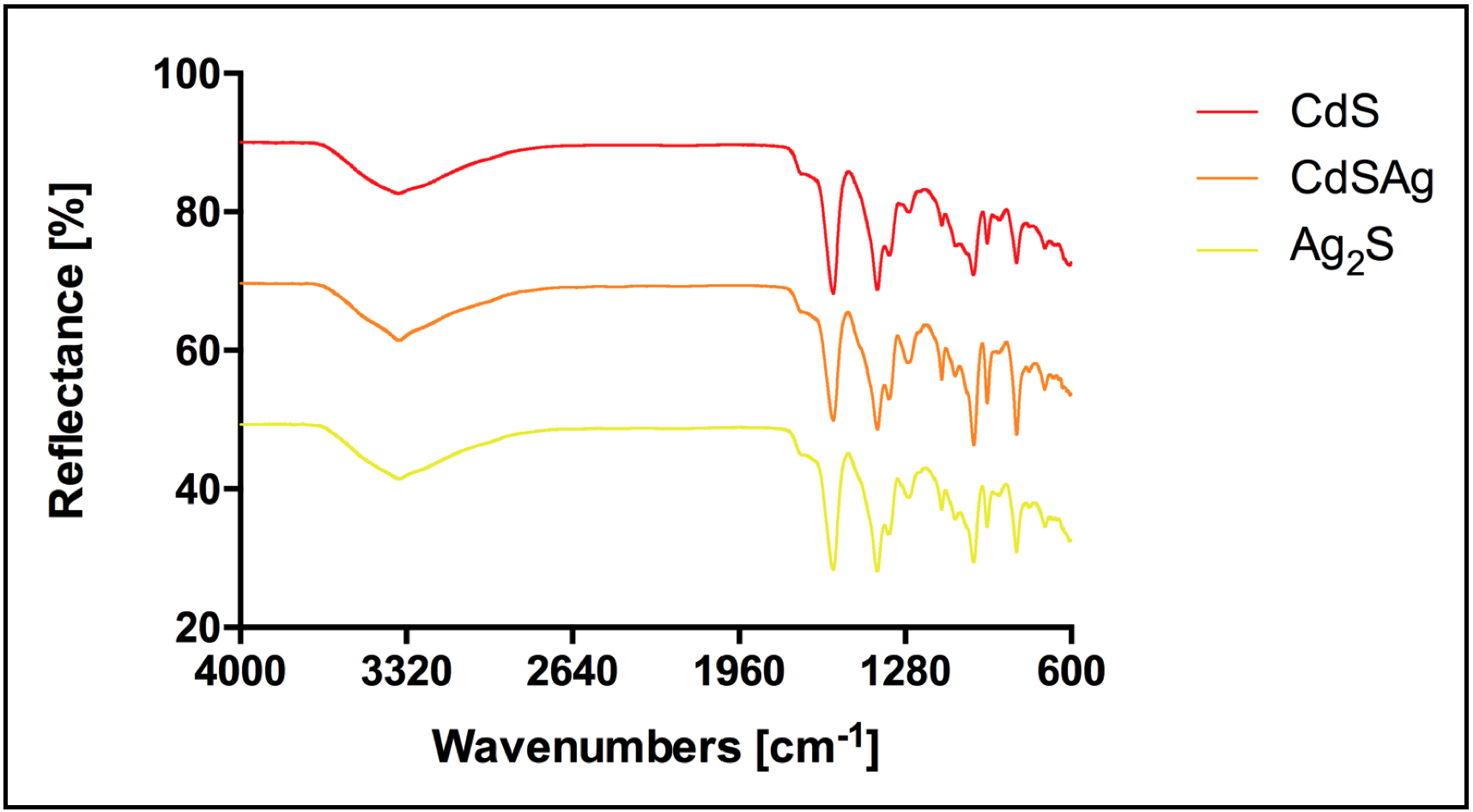

Supplement: Supplementary file 1 [file microorganisms-08-00631-s001.zip › Figura sup 1.tiff]
